# Supplementary material for: Cation-Chloride Cotransporters, Na/K Pump, and Channels in Cell Water and Ion Regulation: In silico and Experimental Studies of the U937 Cells Under Stopping the Pump and During Regulatory Volume Decrease
Source: Front Cell Dev Biol. 2021 Nov 16;9:736488. doi: 10.3389/fcell.2021.736488 (PMC8635019; doi:10.3389/fcell.2021.736488)
Supplement: Supplementary file 1 [file Data_Sheet_1.doc]

How to use the executable file for the program BEZ02BC.

The name and extension of the file DATA SHEET 2.doc must be changed to "DATAB.txt" before use, the name of the file "DATA SHEET 3.doc" must be changed to “BEZ02BC” with extension “exe”. Do not try to open DATA SHEET 3.doc. It is unreadable!

1. The executable file BEZ02BC (BEZ02BC.doc when e-mailed) is best used on a 32-bit

computer with Windows OS. The user should in this case:

a. Locate files DATAB.txt and BEZ02BC with appropriate extension in the same folder.

b. Check the DATAB, the file must correspond to the selected parameters and concentrations, click "Save" (simple “DATAB.txt” without specific name).

c. Run the executable file BEZ02BC with appropriate extension and wait until the process is completed and the file RESB.txt appears.

d. Rename and save the obtained file RESB.txt because in a new running cycle it will be lost.

RESB files can be easily imported by ORIGIN or another program for further processing (the asterisks must be retained).

e. The displayed values of fluxes as well as OSOR correspond to the latest time point. The values of fluxes for other moments can be obtained by setting the necessary time interval with the *hp* value. It is necessary to perform several calculation cycles with a series of the corresponding *hp* to obtain the time course of the fluxes.

f. Some readers of our previous publications have expressed doubt that using our tool it is possible to obtain a unique set of parameters that provide an agreement between experimental and calculated data. Our mathematical comments on this matter can be found in Yurinskaya et al., 2019, P.12.

Several DATAB and appropriate RESB options are presented below as examples:

Example 1 “Iso standard 240”, Cells B, all cotransporters:

DATAB

na0 k0 cl0 B0 kv na k cl beta gamma

140.0 5.8 116.0 48.2 1.0 38.0 147.0 45.0 0.039 1.50

pna pk pcl inc ikc inkcc hp kb

0.00170 0.01150 0.01100 0.000070 0.0000800 0.0000000080 240 0.0

RESB

t U na k cl V/A mun muk mucl naC kC clC

0 -45.0 38.0 147.0 45.0 12.50 -79.9 41.3 19.8 475.0 1837.5 562.5

24 -45.0 38.0 147.0 45.0 12.50 -79.9 41.3 19.8 475.0 1837.5 562.5

....................................................................................

240 -45.0 38.0 147.0 45.0 12.50 -79.9 41.3 19.8 475.0 1837.4 562.4

* na0 k0 cl0 B0 kv na k cl beta gamma

* 140.0 5.8 116.0 48.2 1.000 38.0 147.0 45.0 0.039 1.50

* pna pk pcl inc ikc inkcc hp kb

* 0.00170 0.01150 0.01100 0.0000700 0.0000800 0.0000000080 240 0.000000

* Net_flux PUMP Channel NC KC NKCC

* Na -1.4820 0.4679 1.0171 0.0000 -0.0031

* K 0.9880 -0.5096 0.0000 -0.4753 -0.0031

* Cl 0.0000 -0.5357 1.0171 -0.4753 -0.0061

* Influx PUMP IChannel INC IKC INKCC

* Na 0.0000 0.4927 1.1368 0.0000 0.0874

* K 0.9880 0.1381 0.0000 0.0538 0.0874

* Cl 0.0000 0.4889 1.1368 0.0538 0.1748

* Efflux PUMP EChannel ENC EKC ENKCC

* Na -1.4820 -0.0248 -0.1197 0.0000 -0.0905

* K 0.0000 -0.6477 0.0000 -0.5291 -0.0905

* Cl 0.0000 -1.0246 -0.1197 -0.5291 -0.1809

* z OSOR (A/V)*1000

* -1.75 3.54 80.00

Example 2 “Oua Cells B 960 all cotr.”:

DATAB

na0 k0 cl0 B0 kv na k cl beta gamma

140.0 5.8 116.0 48.2 1.0 38.0 147.0 45.0 0.00 1.50

pna pk pcl inc ikc inkcc hp kb

0.00170 0.01150 0.01100 0.000070 0.0000800 0.0000000080 960 0.0

RESB

t U na k cl V/A mun muk mucl naC kC clC

0 -35.6 38.0 147.0 45.0 12.50 -70.4 50.7 10.3 475.0 1837.5 562.5

96 -22.2 129.0 53.0 55.8 13.86 -24.3 36.9 2.6 1788.6 735.4 774.0

………………………………………………………………..

384 -6.6 165.1 7.8 89.3 20.93 -2.2 1.5 -0.4 3454.5 164.1 1868.6

480 -5.6 164.5 7.3 93.3 22.29 -1.3 0.7 -0.2 3665.9 163.6 2079.5

…………………………………………………………………

864 -4.3 163.6 6.9 98.4 24.31 -0.2 0.1 -0.0 3976.6 166.5 2393.1

960 -4.3 163.5 6.8 98.8 24.46 -0.1 0.1 -0.0 3999.3 166.8 2416.1

* na0 k0 cl0 B0 kv na k cl beta gamma

* 140.0 5.8 116.0 48.2 1.000 38.0 147.0 45.0 0.000 1.50

* pna pk pcl inc ikc inkcc hp kb

* 0.00170 0.01150 0.01100 0.0000700 0.0000800 0.0000000080 960 0.0

* Net_flux PUMP Channel NC KC NKCC

* Na 0.0000 0.0012 0.0062 0.0000 0.0003

* K 0.0000 -0.0002 0.0000 -0.0001 0.0003

* Cl 0.0000 0.0010 0.0062 -0.0001 0.0007

* Influx PUMP IChannel INC IKC INKCC

* Na 0.0000 0.2575 1.1368 0.0000 0.0874

* K 0.0000 0.0722 0.0000 0.0538 0.0874

* Cl 0.0000 1.1768 1.1368 0.0538 0.1748

* Efflux PUMP EChannel ENC EKC ENKCC

* Na 0.0000 -0.2563 -1.1306 0.0000 -0.0871

* K 0.0000 -0.0723 0.0000 -0.0539 -0.0871

* Cl 0.0000 -1.1757 -1.1306 -0.0539 -0.1741

* z OSOR (A/V)*1000

* -1.75 0.00 40.89

Example 3 “Hypo 160 Cells B 240 all cotr.”:

DATAB

na0 k0 cl0 B0 kv na k cl beta gamma

65.0 5.8 41.0 48.2 0.516 38.0 147.0 45.0 0.039 1.50

pna pk pcl inc ikc inkcc hp kb

0.00170 0.01150 0.01100 0.000070 0.0000800 0.0000000080 240 0.0

RESB

t U na k cl V/A mun muk mucl naC kC clC

0 -40.2 19.6 75.9 23.2 24.20 -72.2 28.4 25.0 474.5 1835.7 562.0

24 -41.8 14.1 82.4 19.4 22.67 -82.6 29.1 21.7 319.5 1868.1 439.3

48 -43.4 11.9 85.3 16.8 21.73 -88.8 28.4 19.6 258.1 1854.8 364.6

………………………………………………………………………………………………………………

168 -47.5 10.6 87.8 12.4 20.32 -96.0 25.0 15.6 215.1 1784.9 251.7

192 -47.8 10.6 87.9 12.2 20.27 -96.2 24.8 15.4 214.6 1780.7 247.1

216 -47.9 10.6 87.9 12.1 20.23 -96.3 24.7 15.3 214.4 1778.1 244.2

240 -48.0 10.6 87.9 12.0 20.21 -96.4 24.6 15.2 214.3 1776.4 242.3

* na0 k0 cl0 B0 kv na k cl beta gamma

* 65.0 5.8 41.0 48.2 0.516 19.6 75.9 23.2 0.039 1.50

* pna pk pcl inc ikc inkcc hp kb

* 0.00170 0.01150 0.01100 0.0000700 0.0000800 0.0000000080 240 0.0

* Net_flux PUMP Channel NC KC NKCC

* Na -0.4135 0.2317 0.1776 0.0000 0.0040

* K 0.2757 -0.2171 0.0000 -0.0653 0.0040

* Cl 0.0000 -0.1233 0.1776 -0.0653 0.0080

* Influx PUMP IChannel INC IKC INKCC

* Na 0.0000 0.2381 0.1866 0.0000 0.0051

* K 0.2757 0.1437 0.0000 0.0190 0.0051

* Cl 0.0000 0.1610 0.1866 0.0190 0.0101

* Efflux PUMP EChannel ENC EKC ENKCC

* Na -0.4135 -0.0064 -0.0089 0.0000 -0.0011

* K 0.0000 -0.3609 0.0000 -0.0843 -0.0011

* Cl 0.0000 -0.2843 -0.0089 -0.0843 -0.0021

* z OSOR (A/V)*1000

* -1.75 1.64 49.49

2. To run the executable file on a 64-bit machine, the following additional steps should be taken:

a. Download the School Pak package via Internet and run Norton Commander (NCD).

b. Set in NCD the same folder as the folder in Windows where the DATAB.txt and executable file BEZ02BC are located.

c. Correct the file DATAB.txt if necessary, in the Windows folder.

d. Run executable file in the NCD folder and read RESB.txt in the analogous Windows folder.
